# Discovery of GSK3β Inhibitors through In Silico Prediction-and-Experiment Cycling Strategy, and Biological Evaluation

**DOI:** 10.3390/molecules27123825

**Published:** 2022-06-14

**Authors:** Yuno Lee, Sae-Bom Yoon, Hyowon Hong, Hyun Young Kim, Daeyoung Jung, Byoung-San Moon, Woo-Kyu Park, Sunkyung Lee, Hyukjin Kwon, Jihyeong Park, Heeyeong Cho

**Affiliations:** 1 Drug Information Platform Center, Korea Research Institute of Chemical Technology, 141 Gajeong-ro, Yuseong-gu, Daejeon 34114, Korea; yunolee1@krict.re.kr (Y.L.); leesk@krict.re.kr (S.L.); 2 Therapeutics & Biotechnology Division, Korea Research Institute of Chemical Technology, 141 Gajeong-ro, Yuseong-gu, Daejeon 34114, Korea; bomi9123@krict.re.kr (S.-B.Y.); cnsl428@krict.re.kr (H.H.); aongayo@krict.re.kr (H.Y.K.); dyjeong@krict.re.kr (D.J.); bsmoon@chonnam.ac.kr (B.-S.M.); wkpark@krict.re.kr (W.-K.P.); 3 Department of Biotechnology, Chonnam National University, 50 Daehak-ro, Yeosu 59626, Korea; 4 UNDBIO, 91, Changnyong-Daero 256Beon-gil, Yeongtong-gu, Suwon 16229, Korea; hyukjin.kwon@undbio.com (H.K.); jihyeong.park@undbio.com (J.P.)

**Keywords:** GSK3β, MD simulation, HTRF, pyrazolopyrimidine

## Abstract

Direct inhibitors of glycogen synthase kinase 3β (GSK3β) have been investigated and reported for the past 20 years. In the search for novel scaffold inhibitors, 3000 compounds were selected through structure-based virtual screening (SBVS), and then high-throughput enzyme screening was performed. Among the active hit compounds, pyrazolo [1,5-a]pyrimidin-7-amine derivatives showed strong inhibitory potencies on the GSK3β enzyme and markedly activated Wnt signaling. The result of the molecular dynamics (MD) simulation, enhanced by the upper-wall restraint, was used as an advanced structural query for the SBVS. In this study, strong inhibitors designed to inhibit the GSK3β enzyme were discovered through SBVS. Our study provides structural insights into the binding mode of the inhibitors for further lead optimization.

## 1. Introduction

Glycogen synthase kinase 3 beta (GSK3β) is a multifunctional protein kinase, which shows a broad tissue expression with the highest expression observed in the brain [1,2,3]. It is well known that GSK3β is a key regulator in the signaling pathways of insulin, neurotrophic factor, Wnt/β-catenin and the microtubule-binding protein, tau phosphorylation. Therefore, GSK3β has been suggested as a potential drug target for cancer stem cells and immunological/neurological disorders [4,5]. Researchers have made tremendous efforts to find novel and selective GSK3β inhibitors that can ultimately be applied to drug development. There are hundreds of GSK3β inhibitor-related patents registered, and the main therapeutic areas covered are cancer, neurological diseases, and diabetes [https://www.cortellis.com/drugdiscovery/home?locale=en-US, accessed on 10 May 2022]. Especially for diverse neuropathological aspects, the modulation of GSK3β signaling is an important strategy [6,7,8]. The GSK3β inhibitors in clinical phases are tideglusib (AMO Pharma), Neu-120 (Neurim Pharma) and 9-ING-41 (Actuate Therapeutics) and are under active development as neurologic or anticancer drugs. Recently, it was observed that the inhibition of GSK3β ameliorates the Rett syndrome phenotype and fragile X syndrome in the mouse model ([9,10]).

The catalytic activity of GSK3β is mainly regulated by two phosphorylation sites: autophosphorylation at Tyr216, which activates GSK3β, whereas phosphorylation at Ser9, activated by AKT, decreases its catalytic activity. It has been reported that the active form with phosphorylated Tyr216 is more than half of the total GSK3β in neurons and the brain [11]. GSK3β, with Axin and adenomatous polyposis coli (APC), is the central regulator of the Wnt/β-catenin pathway [12]. Under unstimulated conditions, cytosolic β-catenin is phosphorylated at Ser45 by the priming kinase, casein kinase 1 (CK1). Consequently, GSK3β, in complex with Axin and APC, phosphorylates β-catenin at Thr41, Ser37, and Ser33, inhibiting the Wnt signaling pathway by promoting β-catenin degradation. The inhibition of GSK3β results in the blocking of β-catenin degradation, which can promote β-catenin-induced transcription of neurotrophic factors, such as BDNF [13].

Nitrogenous heterocycles are most commonly used in medicinal chemistry and structural components of pharmaceuticals [14,15,16,17]. Among those, pyrazolopyrimidines are well-known ATP-competitive inhibitors for GSK3β and many kinases, in which the nitrogen of pyrimidine interacts with the carbonyl and NH groups of Val135 residue [18]. Pyrazolo[1,5-a]pyrimidine compounds have also been reported as orally available cyclin-dependent kinase 2 inhibitors [19] and GPR3 inverse agonists [20]. Here, we report on the novel derivatives with pyrazolo[1,5-a]pyrimidine, which are identified as GSK3β inhibitors through a molecular dynamic (MD) simulation, virtual screening, and high-throughput screening.

The Korean Chemical Bank (KCB) is a national chemical depository of synthetic compounds derived from national research projects for over two decades. It holds about 600,000 compounds, of which around 20% are commercially unavailable and unique consigned compounds which are deposited by KRICT’s in-house or national-wide medicinal chemistry researchers. Therefore, we conducted In Silico and experimental screening cycles with these real compounds in the hope that we would find a serendipitous druggable scaffold.

## 2. Results and Discussions

### 2.1. Molecular Dynamics (MD) Simulation of Reference Compound, SB216763, with GSK3β

**Figure 1:**
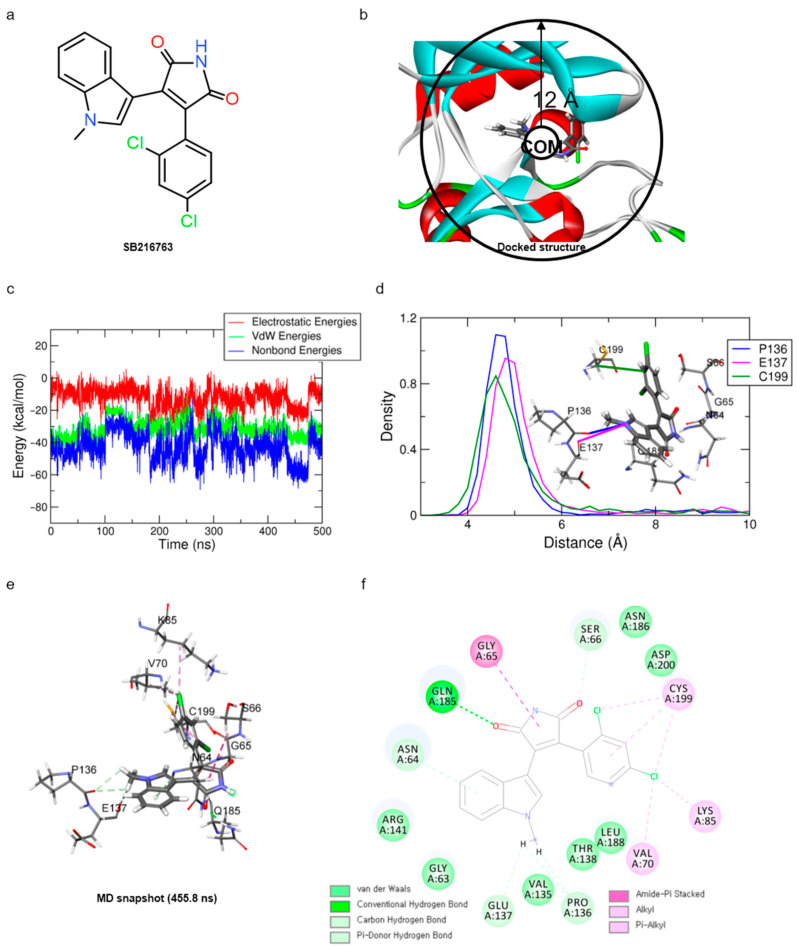
Results of MD simulation for reference compound SB with GSK3β protein. (**a**) Chemical structure of reference compound SB216763. (**b**) Diagram of upper-wall restraint to keep ligand inside the binding site. (**c**) Time traces of protein–ligand non-bonded energies (blue line), which are the sum of electrostatic (red) and vdW (green) energies for SB216763 bound systems. (**d**) Distance distribution of SB216763 with three significantly interacting residues during the final 200 ns. The blue line represents the distance between ligand:N1 and P136:O. The magenta and green lines represent the distance of ligand:N1 with E137:O and ligand:C17 with C199:CB, respectively. The SB216763 is represented as a stick model. (**e**) Binding mode of SB216763 with the ATP-binding site in the GSK3β protein. (**f**) A 2D diagram for the binding mode.

For optimal structure-based virtual screening, it was important to create a reasonable protein–ligand pharmacophore model that could be used as a filtering query. To build this model, the adjusted structure of the binding site to the highly active compound was required. Therefore, SB216763 (Figure 1a, IC_50_ = 34 nM [21]) was docked at the ATP-binding site of the GSK3β protein (PDB ID: 1PYX). In order to find out relatively novel scaffolds rather than mimicking ATP-binding, AMP-PNP was removed from the crystal structure. The initial confirmation of SB216763 was obtained from the molecular docking simulation. Next, we performed MD simulations to create the adjusted protein–ligand complex structure (Figure S1).

Since the MD simulation itself provides the flexibility of both protein and ligand with a solvation effect, the adjustment of the protein–ligand structure was induced with better non-bonded interactions than a molecular docking simulation. Despite these advantages, once the ligand leaves the binding site, it is unable to return to the binding site within a few hundreds of nanosecond time scale. Because the unbinding barrier from the binding site to the solvent region is much lower than the binding barrier where the ligand enters the binding site freely, the ligand is more likely to be in the solvent area.

To overcome this, when the distance between the center of mass (COM) of the binding site residues and the COM of ligand SB216763 is further than 12 Å, the harmonic potential (force constant κ = 200 kJ/mol nm^−2^), by applying a restraint force, was added so that it remains around the binding site to efficiently find a reasonable binding mode (Figure 1b). This force is added only when the distance between the COM of the binding site and ligand is greater than 12 Å, which is the case when the ligand is leaving the binding site.

In order to obtain the proper protein–ligand complex structure, the 500 ns MD simulation was repeated three times (for the first, second, and third trials) under the same system and conditions. The RMSD analysis showed that the system and ligand stabilities for these three trial systems were well stabilized at around 3~5 Å and 0.75~1.25 Å, respectively (Figure S2a,b). Although the different binding mode of SB216763 was observed in the third trial trajectory, the same binding mode was mostly found in the first and second trial trajectories and was maintained over the final 200 ns. Finally, the three-dimensional snapshot of 455.8 ns, showing the most frequent binding mode and having the lowest protein–ligand energy, was extracted from the second trial trajectory (Figure 1c).

For the binding mode analysis of SB216763 with the GSK3β protein, the distance distribution of key residues was calculated during the last 200 ns (Figure 1d). It showed that the carbon–hydrogen bond of SB216763 with the backbone oxygen of P136 and E137, a Pi–alkyl interaction of the ligand with C199, was more important to maintaining the hydrophobic fit into the ATP-binding site than the G65, S66, and Q185 residues that are located in the loop region. (Figure 1e,f) The binding mode was well maintained during the last 200 ns simulation time (Movie S1).

### 2.2. Virtual Screening

**Figure 2:**
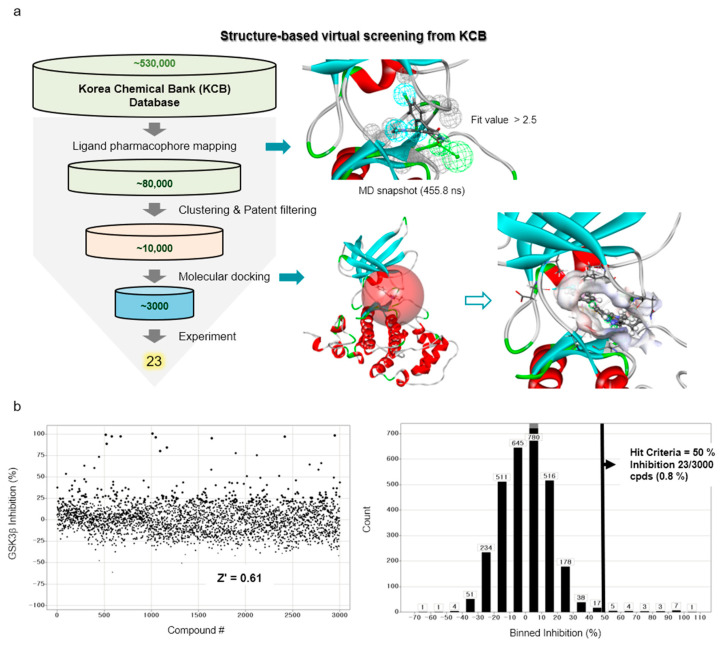
Workflow for the discovery of GSK3β inhibitors using a structure-based virtual screening protocol, including molecular dynamics and molecular docking simulations. (**a**) The procedure of structure-based virtual screening shows the resulted pharmacophore model from the representative structure (in upper right panel) and the calculated binding site (red sphere) with the hit compound, cpd1, for the example of molecular docking simulation (in lower right panel). (**b**) Activity distribution of high-throughput screening campaign.

In order to discover new GSK3β inhibitors from the libraries of Korea Chemical Bank (KCB), we performed a series of structure-based virtual screenings, along with molecular docking and molecular dynamics (MD) simulations (Figure 2a). First, the molecular docking and MD simulations of SB216763 within the ATP-binding site of the GSK3β protein were carried out to obtain the representative structure of the protein–ligand interaction. As mentioned in the previous section, the snapshot of 455.8 ns, which has the most populated binding mode with a favorable non-bond energy, was selected as a representative structure. 

From this structure, a 3D protein–ligand pharmacophore model, comprised of three hydrophobic features and one hydrogen bond acceptor feature, was generated. Then, it was used as a 3D query to retrieve from the KCB database consisting of ~530,000 compounds and then ~80,000 compounds were filtered out through a fit value greater than 2.5. Around 10,000 molecules were selected based on their chemical diversity and patent filtering. These molecules were subjected to a molecular docking simulation and the final top-ranked ~3000 molecules were submitted to an in vitro assay.

### 2.3. HTS Campaign and Following In Vitro Enzyme Assays

The GSK3β inhibitory activities of the virtually-selected 3000 compounds were screened by a Lance HTRF assay. At a concentration of 10 μM, the single-point screening signal-to-noise (S/N) ratio, Z factor, and Z’ factor of the assay was 7.3, 0.48 and 0.61, respectively. The activity distribution of the high-throughput screening campaign was displayed in Figure 2b. Based on a 50% hit criteria, 23 compounds (0.8%) were selected and the IC_50_ values were determined by dose–response experiments.

**Figure 3:**
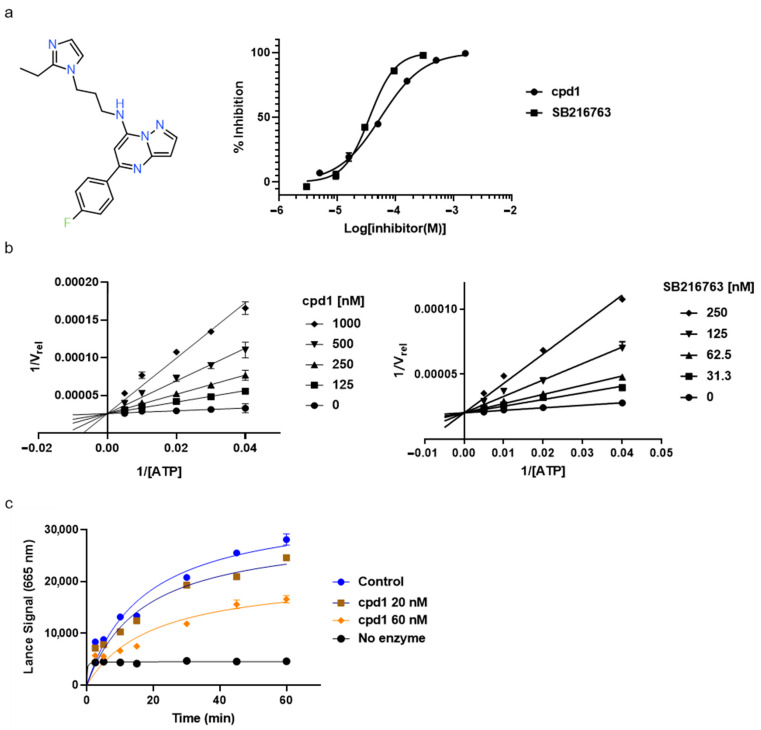
In vitro assay. (**a**) Selected structure of active compound, *N*-(3-(2-ethyl-1H-imidazol-1-yl)propyl)-5-(4-fluorophenyl)pyrazolo[1,5-a]pyrimidin-7-amine (cpd1) and dose–response curve fit of cpd1 and reference SB216763. (**b**) Competitive inhibition by cpd1 and SB216873. Compounds were tested using 4-point 2-fold serial dilution, starting from 1 µM or 0.25 µM at 5 different concentration of ATP (200, 100, 50, 33.3, and 25 µM). (**c**) Reversibility test. GSK3β enzyme was pre-incubated with 1 µM (>20-fold of IC_50_) cpd1 for 30 min and diluted with buffer into indicated concentrations.

Through visual inspection, one compound among the 23 hit molecules was selected, and similar compounds from KCB were searched for. We found around 100 compounds from KCB and measured the IC_50_ values. Finally, five candidate compounds were obtained (Table 1), including the 40 nM hit compound, cpd1 (Figure 3a). Through the five-by-five-concentration kinetic analysis, the inhibition of GSK3β-catalyzed phosphorylation by cpd1 was best described as competitive, with a slope inhibition constant *K* is of 41.8 nM (Figure 3b). Competitive inhibition studies provide information on the specific inhibition pattern and binding potency of inhibitors to the enzyme. Many drugs are competitive inhibitors of the target enzyme, and the IC_50_ (the concentration of the inhibitor that causes a 50% inhibition) values are used to describe the impact of the drugs on enzymes. However, IC_50_ values are affected by the concentration of the substrate, and especially, competitive inhibitors are more dependent on it. Therefore, it is more desirable to determine the *Ki* values based on an enzyme kinetic study using the initial velocity of the linear reaction period. Cpd1 seemed to competitively bind to the same site as ATP. Next, we analyzed the enzyme-binding kinetics of cpd1 using the dilution method, which demonstrated that cpd1 is reversible and ATP-competitive, such as the reference competitive inhibitor, SB216763 (Figure 3c).

### 2.4. Binding Mode Analyses of Hit Compound Cpd1

The molecular docking and MD simulations of the cpd1 were carried out with the protein structure at a 455.8 ns snapshot to obtain the most reasonable binding mode for the hit compound. The initial docking pose of the cpd1 was obtained from the CDOCKER molecular docking simulation. In order to refine the docking pose, an MD simulation of the cpd1 was carried out for 500 ns. The RMSD analysis showed that the system and ligand stability were well stabilized at around 3 Å and 2 Å, respectively (Figure S3a,b).

**Figure 4:**
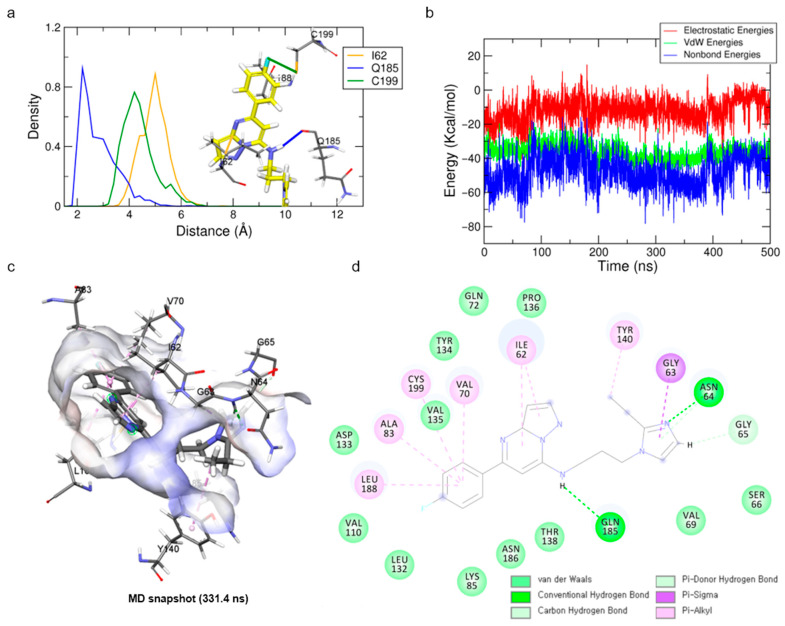
Binding mode results obtained from MD simulation for hit compound cpd1. (**a**) Distance distribution of cpd1 with three key residues during the last 200 ns. The orange, blue, and green lines represent the distance of ligand:C1, ligand:H6, and ligand:F towards I62:CB, Q185:O, and C199:SG, respectively. The cpd1 is shown as a yellow stick model. (**b**) Protein–ligand non-bond energy for cpd1 bound system during the simulation time. (**c**) Binding mode of cpd1 with GSK3β protein. (**d**) A 2D diagram for the binding mode.

For the binding mode analysis of the cpd1, the distance distribution of key residues was calculated during the last 200 ns (Figure 4a). The hit compound, cpd1, showed a hydrogen bond formation with the backbone oxygen of Q185. It also showed a Pi–alkyl interaction with I62, V70, A83, Y140, L188, and C199, and hydrophobic interactions (Figure 4c,d). While the hydrogen bond interaction of Q185 backbone oxygen with the hit compound was stably maintained for the last 200 ns, that of the N64 located in the loop was unstable.

Although the ethyl-imidazole moiety of the cpd1 showed as highly deviated during the simulation time, the core scaffold of pyrazolo[1,5-a]pyrimidine-7-amine with fluorobenzene was stably maintained during the last 200 ns (Movie S2). The snapshot at 331.4 ns, having the lowest protein–ligand energy and highly populated structure, was selected as a representative structure for the cpd1 and used for further study (Figure 4b,c). When the final representative structure was compared with the reference compound, the non-bond energy of cpd1 (−77.95 kcal/mol) was comparable with that of the SB216763 (−68.53 kcal/mol).

### 2.5. Molecular Docking Study for the Hit Derivatives

**Figure 5:**
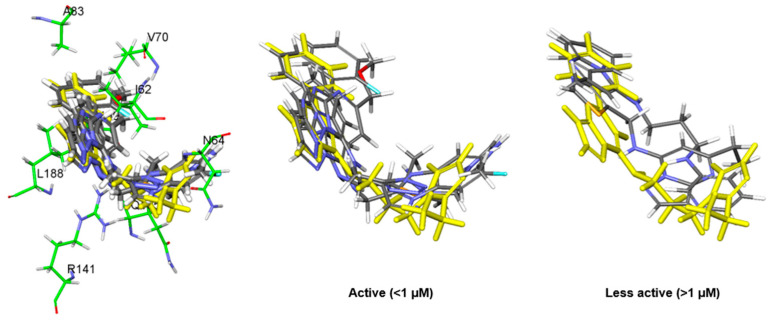
Molecular docking results of hit derivatives. The middle and right panels show overlapped structure of five active molecules which have an IC_50_ less than 1 µM and two less active molecules over than 1 µM, respectively. Hit compound, cpd1, is displayed as a yellow stick model.

To analyze the binding modes of the other hit derivatives, the molecular docking simulations using the CDOCKER and Glide programs were conducted with the representative structure of the cpd1 at 331.4 ns. Since the best correlation coefficient (r = 0.80) of IC_50_ with the docking scores was found in the Glide_energy score (Table 2), the final docked poses from the Glide docking simulation were used for the further pose comparison. In particular, the docked pose of the cpd1 was almost the same (RMSD of 1.08 Å) as that of the cpd1 in the representative structure at 331.4 ns. When we compared the alignment of the core scaffold among the docked conformations of derivatives, five active molecules, which had an IC_50_ of less than 1 μM, were well aligned with each other (Figure 5), showing the linear correlation (r = 0.98) of the Glide_energy values with the experimental IC_50_ values (Table 2).

### 2.6. Wnt Signaling Activity Study for the Active Compounds

**Figure 6:**
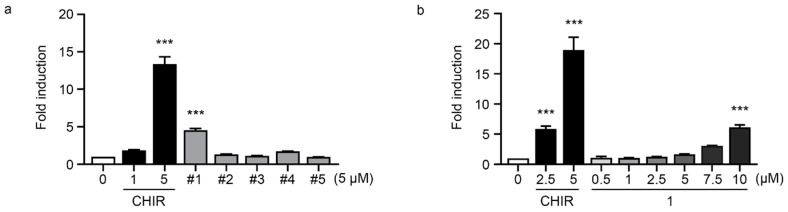
Activation of Wnt signaling pathway by cpd1. (**a**) Reporter analysis using HEK293 TOP flash cell line for measuring activities of GSK3β inhibition on hit compounds. CHIR99021 is a GSK3β inhibitor and used for positive control. Data are mean ± SEM (n = 3). *p*-value by one-way ANOVA (Tukey’s multiple comparisons) test. (**b**) An activation curve for determining the EC50 of cpd1 for TOP flash activity. Data are mean ± SEM (n = 3). *p*-value by one-way ANOVA (Tukey’s multiple comparisons) test. *** *p* < 0.001.

The pharmacologic inhibition of GSK3β’s activity can lead to β-catenin stabilization and subsequently activate Wnt/β-catenin signaling [12]. To measure the inhibitory activities of the compounds selected in GSK3β enzyme-based high-throughput screening, these were treated in HEK293 TOP flash reporter cells at various concentrations. Of the hits, cpd1 showed a significant increase in TOP flash activity (3.5-fold induction at 5 µM) and the induction was dose-dependent (Figure 6).

GSK3β is ubiquitously expressed and constitutively active in unstimulated cells. It is negatively regulated through phosphorylation on Ser9 by AKT, and positively regulated by Tyr216 autophosphorylation, or mediated by the PYK2, MEK1, or SRC kinases [22,23]. Active GSK3β phosphorylates more than 40 proteins, including over 12 transcription factors, and participates in many critical cellular processes in complex ways [4]. GSK signaling is essential for coordinating the proliferation and differentiation of progenitor cells during brain development [2]. However, aberrant hyperactivation of GSK3β and NF-κB is associated with neuroinflammation and many CNS disorders, such as Alzheimer’s disease and epilepsy [24]. In unstimulated (Wnt turned-off) cells, GSK3β phosphorylates β-catenin and downregulates its cellular levels since the phosphorylated β-catenin undergoes subsequent ubiquitination/degradation. When Wnt binds to its heterodimeric receptor (Frizzled receptor andLRP5/6 co-receptor) or is in the presence of GSK3β inhibitors, β-catenin is stabilized and can induce gene transcription. In another aspect, GSK3β and CK-1 phosphorylate LRP5/6 promote Wnt signaling. Although GSK3β has many biological effects, inhibiting GSK3β results in an overall Wnt/β-catenin stimulation and PI3K/PTEN/Akt/mTORC1 pathways. In Figure 3, the selected inhibitors show that cell-based Wnt signaling activation resulted from GSK3β enzyme inhibition.

GSK3β inhibitors can direct neurogenesis and endoderm differentiation [25]. The over-activation of GSK3β is a known link to degenerative brain disorders, diabetes, and cancers [7,8,26]. Therefore, targeting GSK3β has diverse applicability. The virtual simulation and high-throughput screening combination is the most powerful tool in searching for a novel druggable scaffold.

### 2.7. Discussions for Molecular Modeling Approaches

The limitation of the combined study of SBVS with experimental HTS is that the modeling approach is performed with only one target protein, which can be argued whether this approach works for other targets or not. However, based on our previous study [27] and a series of reported papers [28,29], it is generally accepted that an MD simulation improves the existing molecular docking results. In this study, we applied upper-wall restraint force to an MD simulation to reduce the time consumption and increase the chance of obtaining a proper protein–ligand structure. Through this approach, we built the final pharmacophore model and used it for the virtual screening. This strategy was established by referring to our previous studies [30,31] and existing papers [28,29], and with this strategy, it worked in identifying GSK3β inhibitors.

## 3. Materials and Methods

### 3.1. Molecular Docking Simulation

The crystal structure of GSK3β (PDB ID: 1PYX) [32], with a resolution of 2.4 Å, was used as an initial structure for the molecular modeling study. The missing loop regions of the crystal structure were constructed by the MODELER auto model and loop model, implemented in the Discovery Studio (DS) 2018 software [33]. Before proceeding with the molecular docking simulation, all water molecules and ligands were removed, and hydrogen atoms were added to the protein. The GSK3β active site was defined within a radius of 12 Å of the bound 5′-Adenylylimidodiphosphate (AMP-PNP), which is a non-hydrolyzable analog of adenosine 5’-triphosphate (ATP). To generate the initial docking poses subjected to the following molecular dynamics (MD) simulation study, the docking simulations of the SB216763 and hit compounds were performed using CDOCKER [34], implemented in the DS 2018 software. For the purpose of comparison, Schrödinger’s Glide software (version 2019–1, Schrödinger, LLC: New York, NY, USA, 2017) was used for the hit derivatives.

### 3.2. Molecular Dynamics (MD) Simulation

The top-scored docking poses were subjected to the all-atom MD simulations using GROMACS 2016 with the Plumed plugin, version 2.4 [35]. The charmm36 all-atom force field was used for the protein and ligand, and the TIP3P was selected for the explicit solvent model. The CHARMM-GUI [36,37] was used for the generation of input for the simulations. The topologies and parameters of the ligands were generated by the CHARMM General Force Field (CGenFF) program [38]. The fully solvated cubic water box, with a 10 Å thickness, was constructed for each system. NaCl salts (61 Na^+^ and 68 Cl^−^ for the SB216763, and 70 Na^+^ and 77 Cl^−^ for the cpd1 system) were added at a concentration of 0.15 M for neutralizing the system. The systems were energy-minimized by the steepest descent method to remove possible bad contacts until a tolerance of 1000 kJ/mol was reached. The NVT (constant particle number, volume, and temperature) equilibration process was conducted for the minimized structures for 25 ps, with a 1 fs time step at 303.15 K. The LINCS algorithm [39] was used to constrain the bonds involving hydrogen atoms by their equilibrium bond lengths. In total, the four production runs for 500 ns (three repeated runs for SB216763 and one for cpd1) were performed at a temperature of 303.15K, and a pressure of 1 bar by the NPT (constant particle number, pressure, and temperature) dynamics was achieved with the Nosé–Hoover thermostat [40,41] and Parrinello–Rahman [42] barostat. The length of the time step was set to 2 fs for the production runs and the trajectory was saved every picosecond. The cut-off values for the short-range electrostatic interactions and van der Waals interactions were set to 12 Å. The particle mesh Ewald method [43] was used for the long-range electrostatic interactions. To analyze the protein–ligand interactions, a snapshot was selected as a representative structure having the lowest non-bond energy between the protein and ligand with a highly populated conformation of the ligand during the last 200 ns in the second trajectory. In order to avoid the ligand escaping from the binding site to the bulk solvent region, upper-wall restraint force was applied to the system when the distance between the center of mass (COM) of the binding site residues and the COM of the ligand was greater than the cut-off limit (*d_up_*) of 12 Å. For the wall, the harmonic potential was set with a force constant κ = 200 kJ/mol nm^−2^. The restraint force was used as follows with the equation below.

$${\mathrm{Bias}}_{up}=\{\begin{array}{ll} 0 & \mathrm{for}\;\;d<d_{up}\\ k\cdot {\left(d-d_{up}\right)}^{2} & \mathrm{for}\;\;d\;\geq d_{up} \end{array}$$

All the analysis results of the simulations were obtained from GROMACS 2016, DS 2018, and the visual molecular dynamics (VMD), version 1.9.4a12 [44] software.

### 3.3. Pharmacophore Model Generation and Virtual Screening

From the representative structure of the 455.8 ns snapshot for SB216763, the most important pharmacophoric features that are involved in inhibitor binding were extracted by the Interaction Pharmacophore Generation protocol in the DS 2018 software. In order to generate a reasonable size for the pharmacophore model, four to six features in the receptor-ligand pharmacophore generation algorithm were selected. This protocol generates selective pharmacophore models from the features corresponding to the non-bond interactions between the protein and ligand. The Search 3D Database protocol in DS 2018 was used with the generated pharmacophore model to filter out the hit compounds from the ~530,000 compounds in Korea Chemical Bank (KCB). For this search, we used the KCB database, which is an indexed multi-conformer database built by the Build 3D Database protocol in DS 2018. The resulting fit value, clustering, and patent filtering were used for selecting the final ~3000 compounds to submit to an in vitro assay.

### 3.4. High-Throughput Screening (HTS) of GSK3β Kinase Assay (TR-FRET)

The in vitro enzyme TR-FRET (time-resolved fluorescence energy transfer) assays were performed in 384-well white plates (Greiner) at room temperature (25 °C) using an HTS system from Biomek FX (Beckman Coulter) and a Stella robot. The assay was performed in a final volume of 10 µL in a kinase buffer of 50 mM HEPES, pH 7.5, 1 mM EGTA, 10 mM MgCl_2_, 2 mM DTT, and 0.01% Tween-20. The 3000 virtually-selected compounds in the 5 mM DMSO stock were diluted into desired concentrations with kinase buffer. A total of 5 µL of GSK3β (Carna Biosciences, 0.5 ng/well) and 2.5 µL of compounds were added to the assay plates and, then, 2.5 µL of the ULight-GS Peptide (Ser641/pSer657, PASVPPSPSLSRHSSPHQ(pS)ED, #TRF0131 PerkinElmer, 50 nM final concentration)/ATP mixture were added. After 1 h of incubation, the reaction was stopped by adding 10 µL of 24 mM EDTA/Eu-anti-phospho-GS (Ser641) antibody (2 nM, final concentration) mixture. After incubation for 1 h, the fluorescence signal was read in excitation at 320 nm with an emission at 665 nm with the Envision Multilabel Plate Reader (PerkinElmer). Experimental data from dose–response curves were analyzed by nonlinear regression analysis with GraphPad Prism, version 7.00 for Windows, and the IC_50_ values were obtained. For competitive inhibition fits, five-by-five concentrations of ATP and the inhibitor were set and the Lineweaver–Burk transform was used. For the reversibility assay, 20 µL of GSK3β (1 ng/µL) and 20 µL of 20-fold concentrated compounds were pre-incubated for 30 min. A total of 1 µL of the GSK3β/compound mixture and 6.5 µL of kinase buffer were added to the well. Then, 2.5 µL of the Peptide/ATP mixture was added. The reaction was stopped by various incubation times. The values obtained for each compound are designated as the means ± SD (n = 2). A reference GSK3β inhibitor, SB216763, was used in the HTS setup and throughout the whole assays.

### 3.5. Reversibility Assay

A total of 20 µL of GSK3β (1 ng/µL) and 20 µL of the 20-fold concentrated compound were pre-incubated for 30 min. A total of 1 µL of the GSK3β/compound mixture and 6.5 µL of kinase buffer were added to the well. Then, 2.5 µL of the Peptide/ATP mixture was added. The reaction was stopped by various incubation times.

### 3.6. Reporter Assay

The HEK293 reporter cells (HEK293 cells containing the chromosomally incorporated TOPflash gene) were provided by KY Choi (Yonsei University, Seoul, Korea). The HEK293 reporter cells were cultured in DMEM (Gibco) supplemented with 10% FBS (Gibco). To test the activates of GSK3β inhibition for hit compounds, HEK293 reporter cells were seeded into 96-well black polystyrene plates (Greiner Bio-One, Kremsmünster, Austria) at 1.5 × 10^4^ cells per well and cultured for 24 h. Hit compounds, a positive control (CHIR99021, SelleckChem, Houston, TX, USA) or control (DMSO) were added to the wells at the indicated concentrations. After 18 h, the plates were assayed for firefly luciferase activity; relative reporter activity was determined after the data were normalized relative to the value of the WST-1 (Takara Korea Biomedical Inc., Seoul, Korea) cell viability analysis. Statistical analyses were performed using Prism 8 (GraphPad Software). A one-way ANOVA (Tukey’s multiple comparisons) test (* *p* < 0.05, ** *p* < 0.005, *** *p* < 0.001) was used to test hypotheses about the effects in multiple groups.

## 4. Conclusions

A systematic structure-based virtual screening process, including molecular docking and the upper-wall restrained MD simulations, was engaged to identify hit compounds. In this study, the binding features obtained from the MD simulation of the reference compound (SB216763) were used to identify the candidate compounds that bind at the ATP-binding site. Through the molecular modeling study of the final hit compounds, the proper binding mode of the core scaffold, pyrazolo[1,5-a]pyrimidine-7-amine, was identified with good agreement with the experimental IC_50_ values. These hit compounds showed a higher number of hydrogen bond interactions than the reference compound and more hydrophobic interactions. Taken together, our findings suggest five candidate compounds as novel GSK3β inhibitors.

## Figures and Tables

**Table 1:**
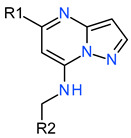
GSK3β inhibitory effects of derivatives of pyrazolo[1,5-a]pyrimidine.

Serial Number	R^1^	R^2^	GSK3β Kinase Assay	
			%Inhibition (0.5 μM/5 μM)	IC_50_ (nM)
**1**	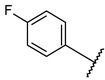	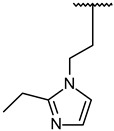	100/100	37 ± 6
**2**	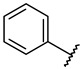	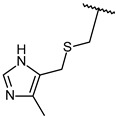	78/100	211 ± 55
**3**	Et	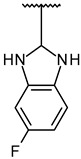	57/98	379 ± 50
**4**	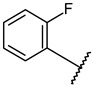	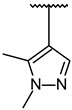	57/96	347 ± 56
**5**	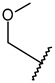	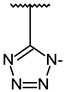	38/95	701 ± 118
**6**	Et	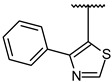	45/91	730 ± 113
**7**	CH_3_	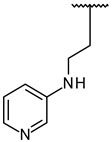	30/90	1027 ± 66
**8** (SB216763)			61/100	35 ± 12

**Table 2:** Binding energy scores of Glide, negative CDOCKER energy (CE), negative CDOCKER interaction energy (CIE) obtained from molecular docking simulations for hit derivatives.

Compound	IC_50_ (nM)	Glide_Evdw	Glide_Ecoul	Glide_Energy	Glide_XP_Hbond	CIE	CE
**1**	40	−43.94	−7.05	−50.99	−1.08	45.67	21.48
**2**	254	−40.66	−7.07	−47.73	−0.35	41.11	10.56
**3**	334	−33.39	−7.56	−40.95	−0.97	33.25	8.16
**4**	485	−39.62	−2.47	−42.09	−0.31	34.49	−56.24
**5**	776	−30.95	−4.09	−35.04	−0.40	39.27	−3.75
**6**	1166	−40.90	−2.14	−43.04	−0.03	34.04	10.12
**7**	1190	−33.86	−3.85	−37.71	0	33.13	13.95

## Data Availability

Not applicable.

## Supplementary Materials

The following supporting information can be downloaded at: https://www.mdpi.com/article/10.3390/molecules27123825/s1, Figure S1: Flow chart of molecular modeling approach for generating the reasonable protein-ligand pharmacophore model; Figure S2: Protein C-alpha and ligand RMSD of three trial systems for binding of SB216763; Figure S3: Protein C-alpha and ligand RMSD results obtained from MD simulation of hit compound, cpd1; Video S1: MD trajectory for binding event of SB216763 during the last 200 ns (300~500 ns); Video S2: MD trajectory for binding of hit compound, cpd1 during the last 200 ns.

## Sample Availability

Samples of the compounds are available from the authors.
