# Supplementary material for: Discovery of GSK3β Inhibitors through In Silico Prediction-and-Experiment Cycling Strategy, and Biological Evaluation
Source: Molecules. 2022 Jun 14;27(12):3825. doi: 10.3390/molecules27123825 (PMC9230645; doi:10.3390/molecules27123825)

Supplementary Materials

Supplementary Materials include:

Supplementary figures, movies and related legends

Supplementary Figure S1 | Flow chart of molecular modeling approach for generating the reasonable protein-ligand pharmacophore model

Supplementary Figure S2 | Protein C-alpha and ligand RMSD of three trial systems for binding of SB216763

Supplementary Figure S3 | Protein C-alpha and ligand RMSD results obtained from MD simulation of hit compound, cpd1

Supplementary Movie S1 | MD trajectory for binding event of SB216763 during the last 200 ns (300~500 ns)

Supplementary Movie S2 | MD trajectory for binding of hit compound, cpd1 during the last 200 ns

Supplementary Figure legends

Supplementary Figure S1 | Flow chart of molecular modeling approach for generating the reasonable protein-ligand pharmacophore model. a, A series of conducted simulations including molecular docking and molecular dynamics to generate 3D structure-based pharmacophore model. **b**, Comparison of obtained structures from molecular docking (left) and molecular dynamics (right) simulation for SB216763. Superimposed structure between them is shown in the middle panel. The initial structure of SB216763 is represented as yellow stick model.

Supplementary Figure S2 | Protein C-alpha and ligand RMSD of three trial systems for binding of SB216763. a, Root mean square deviation (RMSD) of C-alpha atoms of the GSK3β protein from three trial (1st trial with black line, 2nd red, 3rd green) MD simulation trajectories. **b**, Ligand RMSD of SB216763 from the three trials.

Supplementary Figure S3 | Protein C-alpha and ligand RMSD results obtained from MD simulation of hit compound, cpd1. a, C-alpha RMSD of the GSK3β protein obtained from the MD simulation of the protein with hit compound, cpd1. **b**, Ligand RMSD of the cpd1 from the simulation.

Supplementary Movie S1 | MD trajectory for binding event of SB216763 during the last 200 ns (300~500 ns). The SB216763 is displayed as thick stick model and interacting residues are represented as thin stick model. The three key residues (P136, E137, and C199) are only colored by atom name. Due to the thermal fluctuations, the trajectory frames were smoothed with a window size of 5 frames.

Supplementary Movie S2 | MD trajectory for binding of hit compound, cpd1 during the last 200 ns. The cpd1 is represented as thick stick model and interacting residues are thin stick model. The three key residues (I62, Q185, and C199) are only colored by atom name.

Supplementary Figure S1.

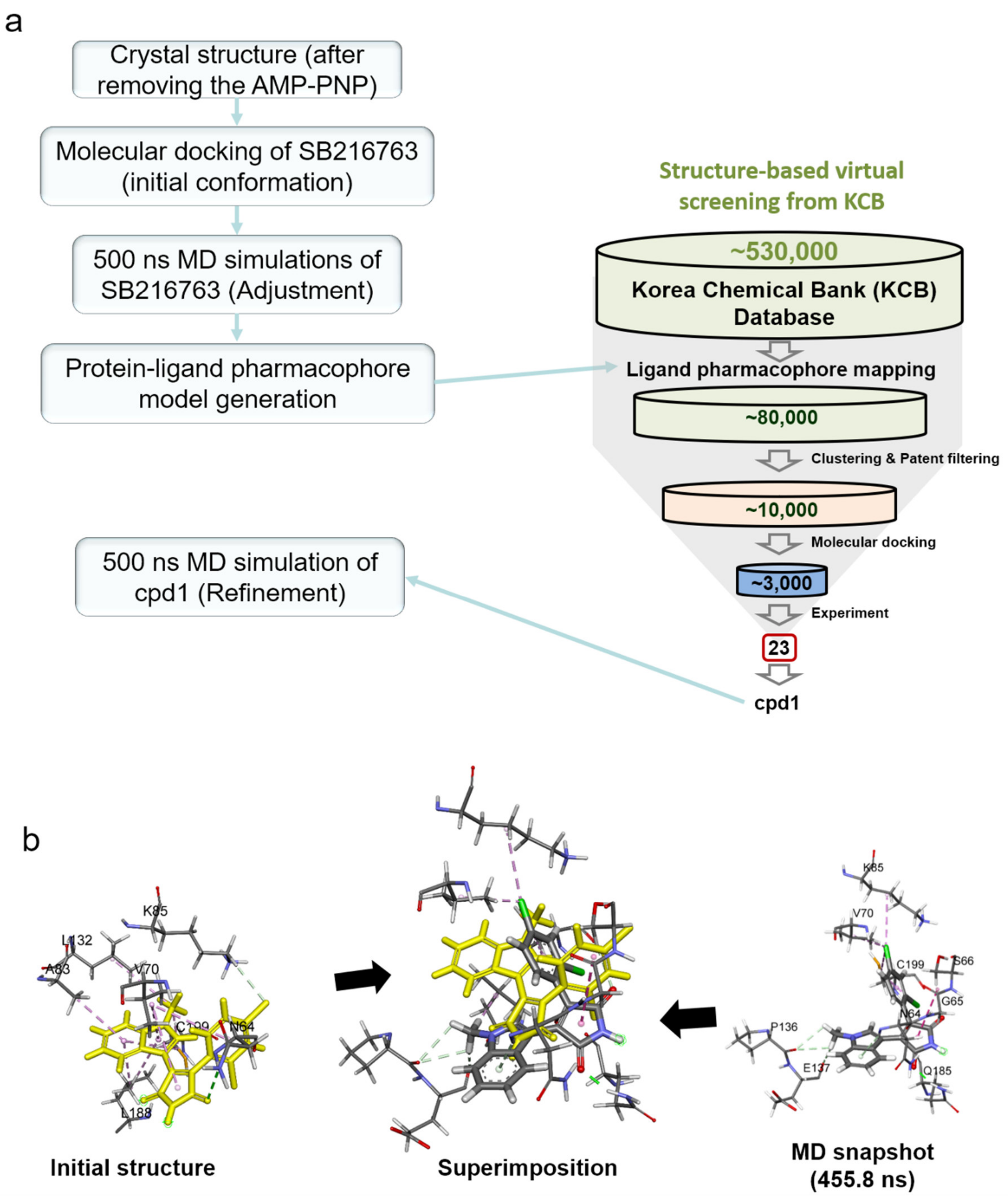

Supplementary Figure S2.

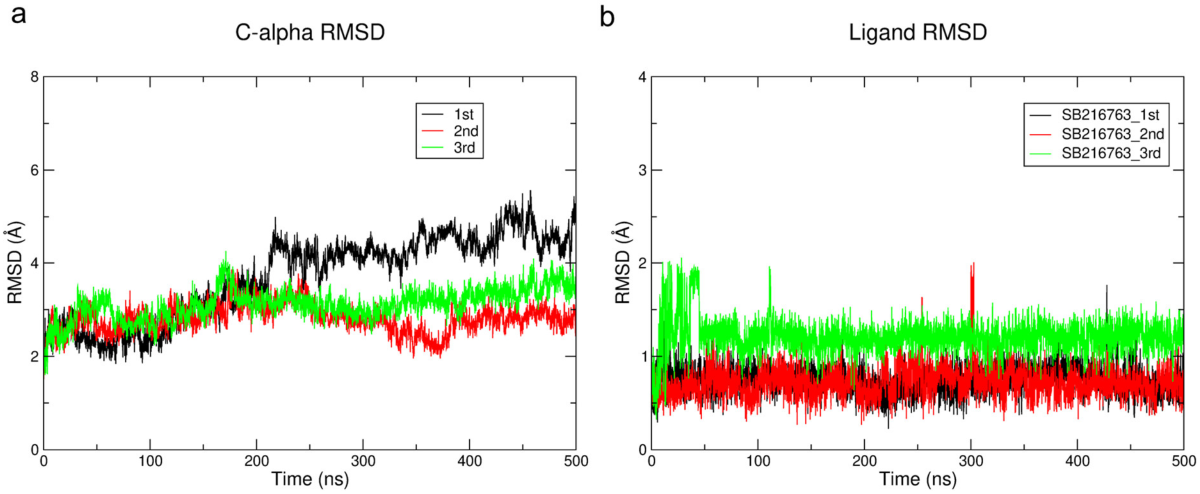

Supplementary Figure S3.

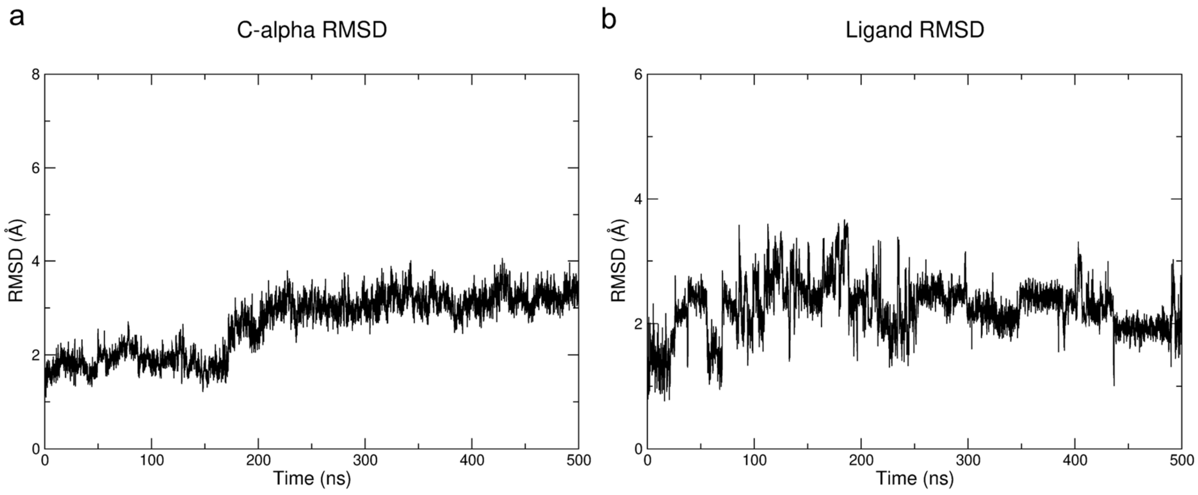

Supplementary Movie S1.

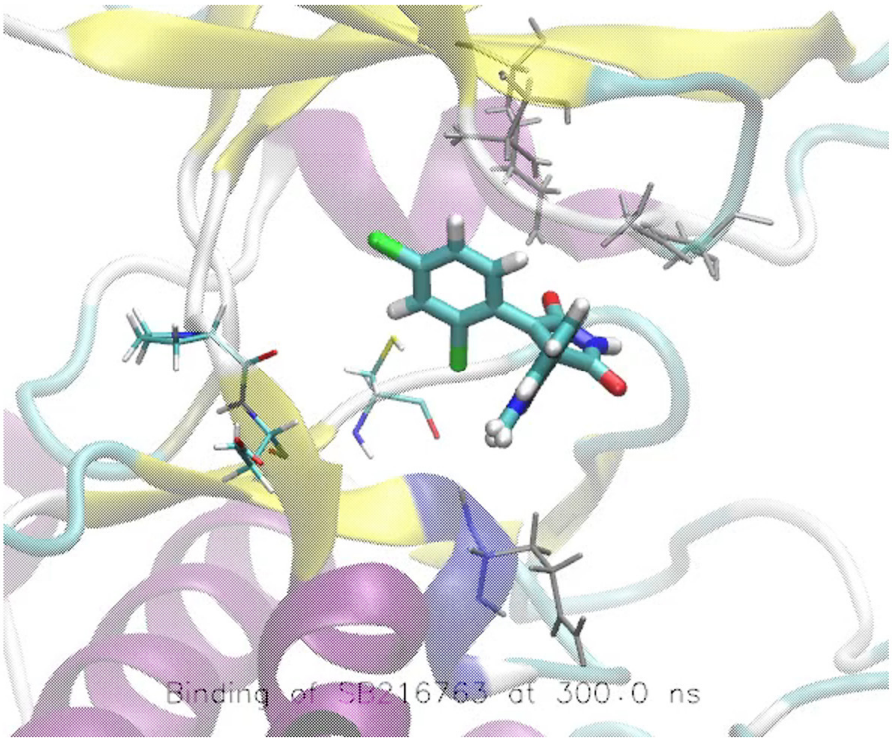

Supplementary Movie S2.

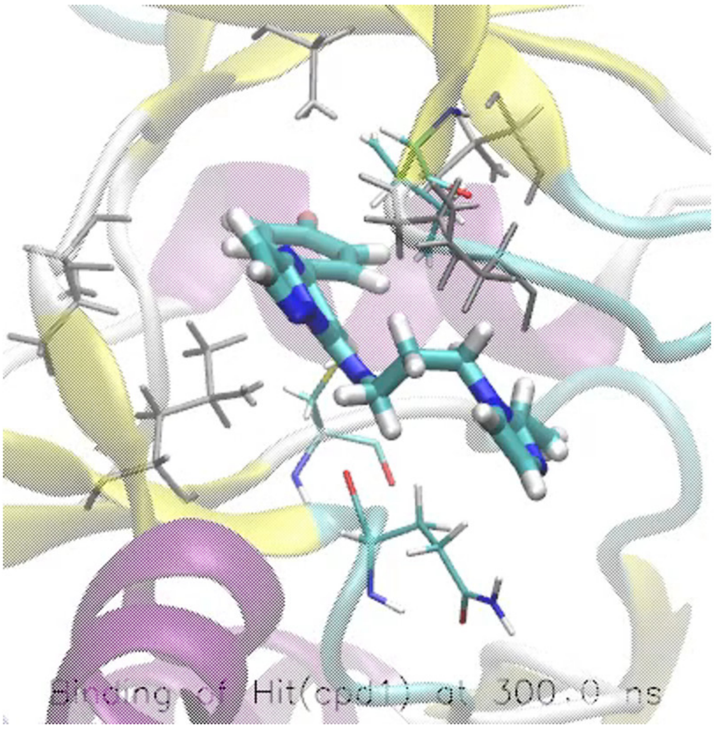

Supplement: Supplementary file 1 [file molecules-27-03825-s001.zip › Supplementary_Figures_and_Movies_2022_0608.pdf]
